# The Effect of NNK, A Tobacco Smoke Carcinogen, on the miRNA and Mismatch DNA Repair Expression Profiles in Lung and Head and Neck Squamous Cancer Cells

**DOI:** 10.3390/cells9041031

**Published:** 2020-04-21

**Authors:** Sotirios G. Doukas, Dimitra P. Vageli, George Lazopoulos, Demetrios A. Spandidos, Clarence T. Sasaki, Aristidis Tsatsakis

**Affiliations:** 1 Department of Forensic Sciences and Laboratory of Toxicology, Medical School, University of Crete, 71003 Heraklion, Greece; sotirios.doukas.mail@gmail.com (S.G.D.); tsatsaka@uoc.gr (A.T.); 2 Department of Surgery, The Yale Larynx Laboratory, New Haven, CT 06510, USA; clarence.sasaki@yale.edu; 3 Department of Cardiothoracic Surgery, Medical School, University of Crete, 71110 Heraklion, Greece; g.lazopoulos@med.uoc.gr; 4 Laboratory of Clinical Virology, Medical School, University of Crete, 71110 Heraklion, Greece; spandidos@spandidos.gr

**Keywords:** tobacco smoke, NNK, head and neck cancer, lung cancer, Mismatch DNA repair, MSH2, MLH1, miR-21, miR-155, miR-422a

## Abstract

Tobacco smoking is a common risk factor for lung cancer and head and neck cancer. Molecular changes such as deregulation of miRNA expression have been linked to tobacco smoking in both types of cancer. Dysfunction of the Mismatch DNA repair (MMR) mechanism has also been associated with a poor prognosis of these cancers, while a cross-talk between specific miRNAs and MMR genes has been previously proposed. We hypothesized that exposure of lung and head and neck squamous cancer cells (NCI and FaDu, respectively) to tobacco-specific nitrosamine 4-(methylnitrosamino)-1-(3-pyridyl)-1-butanone (NNK) is capable of altering the expression of MSH2 and MLH1, key MMR components, by promoting specific miRNA deregulation. We found that either a low (1 μM) or high (2 μM) dose of NNK induced significant upregulation of “oncomirs” miR-21 and miR-155 and downregulation of “tumor suppressor” miR-422a, as well as the reduction of MMR protein and mRNA expression, in NCI and FaDu, compared to controls. Inhibition of miR-21 restored the NNK-induced reduced MSH2 phenotype in both NCI and FaDu, indicating that miR-21 might contribute to MSH2 regulation. Finally, NNK exposure increased NCI and FaDu survival, promoting cancer cell progression. We provide novel findings that deregulated miR-21, miR-155, and miR-422a and MMR gene expression patterns may be valuable biomarkers for lung and head and neck squamous cell cancer progression in smokers.

## 1. Introduction

Plenty of epidemiologic data have shown the association of tobacco smoking with the development of human malignancies [1,2]. Although the antismoking campaign in multiple countries has shown positive results, the rate of smokers in both the United States and Europe remains significant [3,4,5]. It is estimated that nearly 40 percent of diagnosed cancers are directly or indirectly related to tobacco smoking [6].

Lung cancer has the highest mortality rates in the world for both men and women, and is the most common type of malignancy in men [7]. In addition, head and neck cancer, and especially head and neck squamous cell carcinoma (HNSCC), represents one of the most aggressive malignancies with a high rate of mortality. [8,9]. Tobacco smoking has been one of the most well-established risk factors for both lung and head and neck cancers [1,2,3,4,5,6,7,8]. It is known that tobacco smoke contains a mixture of thousands of compounds, including a large number of known carcinogens [2]. It is believed that exposure of cells to tobacco smoke carcinogens can lead to DNA damage, which may cause chromosomal instability and increased cell proliferation [10,11,12,13]. In particular, tobacco-specific nitrosamine 4-(methylnitrosamino)-1-(3-pyridyl)-1-butanone (NNK), which is one of the chemicals in tobacco smoke, has been linked to lung and head and neck cancer [14], and has also been shown to upregulate oncogenic pathways [15,16,17].

The development and progression of lung and head and neck malignancies appear to be a complex process. Although multiple diagnostic and prognostic markers have been identified for both lung and head and neck cancers [18,19], the precise molecular mechanisms involved in the development and progression of these malignancies remain unclear.

We understand that a functional DNA repair mechanism that includes the recognition and repair of mismatch DNA errors during DNA replication is essential in eliminating the harmful effect of several environmental risk factors, such as NNK, on the exposed cells [20,21,22,23]. A number of studies have shown that reduced expression of mismatch DNA repair (MMR) genes increases the incidence of microsatellite instability [24,25,26,27], which is often found in head and neck cancer [28,29,30,31]. Other studies have also shown that reduced expression of *MSH2* or *MLH1* genes at the protein or mRNA levels is associated with poor survival and MSI in lung cancer [32,33,34].

In addition, MMR deficiency appears to affect the effectiveness of chemotherapy in these cancers [34,35]. Also, MMR status has been shown to influence the effectiveness of target immunotherapy, including PD-1 and PD-L1 inhibitors, for lung and head and neck cancers [36]. Therefore, several studies have focused on the assessment of the MMR status, as this may have a significant predictive value for these patients. [23,24,34,36,37].

A number of regulatory molecules such as miRNAs have been suggested to be implicated in the regulation of MMR genes [38,39,40,41,42,43,44,45,46]. In particular, recent studies support a cross-talk between specific miRNAs and MMR genes [41,42,43]. It has been suggested that tumor suppressor miRNA-422a plays an important regulatory role in MLH1 expression, which is responsible for repairing DNA damage [44]. Some reports have also shown that oncomir miR-21 downregulates *hMSH2* gene expression by targeting the 3′ untranslated region of its mRNA [45], and that miR-155 can significantly downregulate *hMSH2, hMSH6*, and *hMLH1* [46], while others have suggested that miRNAs play an important role in modulating cell cycle progression by targeting *hMSH2* in lung cancer [42]. Although there are reports suggesting a relationship between the MMR mechanism and miRNA profiles [41,43,44,46], the underlying molecular mechanism by which tobacco smoke carcinogens induce miRNA deregulation and affect the expression profiles of mismatch repair genes, particularly in lung and head and neck cancer, is not yet known.

Here, we attempt to explore whether NNK affects the expression of small regulatory molecules, such as known miRNA markers, previously associated with upper aerodigestive tract malignancies [47,48,49,50,51,52,53,54] that may directly or indirectly be involved in the regulation for MMR expression phenotypes. Understanding the molecular changes induced by various risk factors, such as tobacco smoke, which promote the development and progression of cancer, will help to develop new diagnostic and therapeutic approaches [55,56], leading to optimization of their management.

## 2. Materials and Methods

### 2.1. Cell Culture and Treatment Conditions

#### 2.1.1. Human Hypopharyngeal and Lung Squamous Cancer Cell Culture

Human hypopharyngeal squamous cancer cells (HSCC), FaDu (HTB-43), were provided by ATCC, Manassas, VA, USA, and cultured in Eagle’s Minimum Essential Medium (EMEM, ATCC, Manassas, VA, USA), 10% FBS, 1% pen/strep, at 37 °C in humidified air and 5% CO_2_. Human lung squamous cancer cells (LSCC), NCI (NCI-H1703), were provided by ATCC, Manassas, VA, USA, and cultured in RPMI-1640 medium (ATCC, Manassas, VA, USA) 10% FBS, 1% pen/strep, at 37 °C in humidified air and 5% CO_2_.

#### 2.1.2. Treatment Conditions

Cancer cells reached 70–80% confluency and were then exposed to experimental media for 24 h. Experimental groups included exposure to (i) 1 μΜ and (ii) 2 μΜ of 4-(*N*-Methyl-*N*-Nitrosamino)-1-(3-pyridyl)-1-butanone (CAS 64091-91-4) or NNK (sc-209854 Santa-Cruz^®^, Dallas, TX, USA) [17] (NNK dissolved in DMSO-vehicle as 1 M stock solution). Cells were incubated in serum-free medium (EMEM for FaDu and RPMI-1640 for NCI, with 1% pen/strep) with NNK, at 37 °C in humidified air and 5% CO_2_. The untreated control groups for each cancer cell line, NCI and FaDu, consisted of cells incubated in serum-free media (EMEM or RPMI-1640, 1% pen/strep, with the vehicle but without NNK). Experimental and control groups were cultured in parallel for each cancer cell line. All the experiments were independently repeated three times. Cells were harvested at the end of the treatment cycle.

### 2.2. Immunoperoxidase Cell Staining for MSH2

We performed an immunocytochemical analysis to detect nuclear and cytoplasmic MSH2 levels in NCI and FaDu cells exposed to NNK relative to untreated controls. Specifically, NCI and FaDu were grown on slides (multiwall chamber slides; Nunc^TM^ Lab-Tek^TM^, Thermo Scientific, Waltham, MA, USA) and treated with experimental media of 1 μM or 2 μM NNK. Untreated controls were also used for each cancer cell line.

Cells were fixed immediately after the final exposure to experimental or control media in 4% paraformaldehyde for 10 min, followed by 3 washes with PBS and permeabilization of the cell membranes using 0.02% Triton X100-PBS (AmericanBio, Natick, MA, USA) for 3 min. Cells were incubated with 0.5% H_2_O_2_ in PBS for 10 min, followed by 2 washes with PBS and blocking with 5% bovine serum albumin (BSA)-PBS (Sigma-Aldrich, St. Louis, MO, USA) for 1 h, and incubation with 1:100 primary mouse-monoclonal antibody HRP for DNA mismatch repair protein MSH2 (MutS protein homolog 2, MSH2_HUMAN, Clone D-6, Santa Cruz Biotechnology, Inc., Dallas, TX, USA), for 1 h at 37 °C. Cells were washed in 1% Tween-PBS and incubated to 1:100 dilutions of secondary mouse-IgGκ BP-HRP (Santa Cruz Biotechnology Inc., Dallas, TX, USA), for 30 min at 37 °C. Subsequently, cells were incubated for 1 min with freshly mixed DAB (3,3′ Diaminobenzidine Tetrahydrochloride in 0.1 M Tris-HCl pH 7.6) with 0.5% hydrogen peroxide, washed in distilled H_2_O and counterstained with Gill’s Hematoxylin Solution, No. 2 (Santa Cruz Biotechnology Inc., Dallas, TX, USA) for 5 s. Finally, cells were washed with several changes of distilled H_2_O and dehydrated in graded alcohols, cleared with xylene and mounted with permanent mounting medium and coverslip.

Slides were examined using a Leica light microscope and images were captured by Aperio CS2 and analyzed by Image Scope software [57]. Expression levels of nuclear MSH2, in experimental and control NCI and FaDu groups, were assigned as positivity [Np/Nt = Number of nuclear positive/total number of nuclei, means (SD)] from at least two independent images (≥10 cells).

### 2.3. Immunofluorescence Cell Staining for MLH1

We performed an immunofluorescence assay to detect nuclear and cytoplasmic MLH1 levels in NCI and FaDu cells exposed to NNK relative to untreated controls. Specifically, NCI and FaDu were grown on slides (multiwall chamber slides; Lab-Tek^®^) and treated with experimental media of 1 μM and 2 μM NNK. Untreated controls were also used for each cancer cell line.

Cells were fixed immediately after the final exposure to experimental or control media in 4% paraformaldehyde for 10 min, followed by 3 washes with PBS and permeabilization of cell membranes using 0.02% Triton X100-PBS (AmericanBio, Natick, MA, *USA*) for 3 min, blocking with 2% bovine serum albumin (BSA)-PBS (Sigma-Aldrich) for 1 h, and incubation with 1:50 of primary mouse-monoclonal antibody for DNA mismatch repair protein MLH1 (MutL protein homolog 1, MLH1 HUMAN, Clone A-8, Santa Cruz Biotechnology Inc., Dallas, TX, USA), overnight at 4 °C. Cells were washed in 1% Tween-PBS and incubated with 1:500 dilutions of secondary antimouse DyLight^®^488 (green; Vector Labs, Burlingame, CA, USA), for 1 h, at room temperature. Finally, cells were mounted using Prolong Gold Mountant with diamidino-phenylindole (ProLong^®^ Diamond Antifade Mountant with DAPI; Life Technologies, Thermo Scientific, Franklin, MA, USA) for nuclear staining of cells (blue).

Slides were examined using a Zeiss Confocal microscope and images were captured and analyzed using the Zen imaging software from Carl Zeiss, microscopy, Germany, as previously described [58,59]. Expression levels of nuclear MLH1, in experimental and control NCI and FaDu groups, were identified by fluorescence intensity [MLH1/DAPI means(SD) bin count] from four independent images (≥10 cells) (Zen imaging software, Carl Zeiss, microscopy GmbH, Jena, Germany).

### 2.4. Western Blotting for MMR Proteins

We performed a Western blot analysis to determine the expression levels of MSH2 and MLH1 nuclear and cytoplasmic proteins in experimental and control NCI and FaDu groups. We used β-actin and Histone 1 to normalize cytoplasmic and nuclear extracts, respectively, and expression levels were estimated using the Image Lab 5.2 analysis software (Bio-Rad, Hercules, CA, USA), as previously described [59,60]. Specifically, 20 to 30 µg of nuclear and cytoplasmic NCI and FaDu protein extracts were heated at 70 °C for 10 min in sodium dodecyl sulfate-polyacrylamide gel electrophoresis Laemmli sample buffer (Bio-Rad, Hercules, CA, USA), and separated using 420–% Mini-PROTEAN TGX Tris/Glycine precast gels, at 150V for 1 h. We used precision plus prestained protein standards (Dual Color or Kaleidoscope, *BIO-RAD*) as molecular-weight size markers. Proteins were transferred onto a 0.45 mm nitrocellulose membrane, using a Trans-Blot Turbo transfer system (Bio-Rad). After blocking in 5% BSA, for 1 h, membranes were incubated with 1:300 primary antibodies, MSH2 (Clone D-6) and MLH1 (Clone A-8) (Santa Cruz Biotechnology Inc., Dallas, TX, USA), which were diluted in 5% BSA, overnight at 4 °C. Membranes were incubated for 1:30 h with goat antimouse horseradish peroxidase-conjugated secondary antibodies (EMD Millipore, Burlington, MA, USA) at 1:2000, and chemiluminescence was determined using an enhanced chemiluminescence detection system (Clarity Western ECL Substrate, Bio-Rad). Membranes were also stripped using Restore^TM^ Western Blot Stripping buffer (Pierce Biotechnology, Rockford, IL, USA) and reported with β-actin (C4; Santa Cruz Biotechnology Inc., Dallas, TX, USA) for cytoplasmic extracts and Histone 1 (AE-4; Santa Cruz Biotechnology Inc., Dallas, TX, USA) for nuclear extracts normalization. Protein levels were quantified by the Gel imaging system (Bio-Rad, Hercules, CA, USA) in each nuclear or cytoplasmic cellular compartment (Image Lab 5.2 analysis software, Bio-Rad, Hercules, CA, USA).

### 2.5. Quantitative Real-Time PCR for hMSH2 and hMLH1

We used a real-time quantitative polymerase chain reaction (qPCR) analysis to evaluate the transcriptional levels of *hMSH2* and *hMLH1*. Total RNA (RNeasy mini kit; Qiagen Inc., Valencia, CA, USA) was isolated from NCI and FaDu exposed to 1 μM or 2 μM of NNK and their corresponding untreated controls. Briefly, we determined RNA quality and concentration by absorption ratios at 260/280 nm (>2.0) and 260 nm, respectively (NanoDrop^TM^ 1000 spectrophotometer; Thermo Fisher Scientific, Waltham, MA, USA). We performed reverse transcription (iScript cDNA synthesis kit; Bio-Rad) from total RNA and real-time qPCR analysis (Bio-Rad real-time thermal cycler CFX96^TM^; Bio-Rad) using specific primers for target genes and reference housekeeping gene, human glyceraldehyde 3-phosphate dehydrogenase (*h*GAPDH) (QuantiTect Primers Assays; Qiagen) (Supplementary Table S1), and iQ^TM^ SYBR Green Supermix (Bio-Rad). We performed assays on 96-well plates, in triplicate for each sample, and data were analyzed using the CFX96^TM^ software [59,60]. Relative mRNA expression levels were estimated for each target gene compared to the reference control gene (ΔΔ*C*t).

### 2.6. miRNA Analysis for miR-21, miR-155 and miR-422a

We performed a miRNA analysis by qPCR to show the expression of “oncomirs” *miR-21* and *miR-155*, and “tumor suppressor” *miR-422a*, and to monitor the effect of 1 μM and 2 μM of NNK on NCI and FaDu treated cells, compared to their corresponding untreated controls. We used miScript II RT kit (Qiagen) to perform reverse transcription synthesis of miRNAs from total RNA (isolated for miRNA analysis as described above) according to the manufacturer’s instructions, using specific primers for target miRNAs of the human genome and normalization control small RNA RNU6B (snRNA *RNU62–*) (miScript Primer Assay; miScript SYBR Green PCR Kit; Qiagen, Louisville, KY, USA) (Supplementary Table S2), as previously described [57,58]. The relative expression levels (target miRNA/RNU6B) for each specific miRNA marker were assessed in each NCI and FaDu group treated with NNK and their untreated controls, by CFX96^TM^ software (Bio-Rad).

### 2.7. Transfection of NCI and FaDu with Mimic/Inhibitor of miR-21

MicroRNA-21 mimic (miScript miRNA Mimic, Qiagen) or miR-21 inhibitor (Antihsa-miR-21-5p; Qiagen) (Supplementary Table S1) were diluted to a final concentration of 5 nM in serum-free culture medium, including HiPerFect^®^ Transfection Reagent (3μL/well) (Qiagen), according to the manufacturers’ instructions, and incubated for 10 min at room temperature. Cells (NCI and FaDu) were mixed with transfected complexes, seeded at 1.5 × 10^5^ cells/well of 24-well plates and incubated for 24 h under normal growth conditions (at 37 °C and 5% CO_2_). The next day, the media were removed and replaced with experimental media of 1 μM or 2 μM NNK. Untreated control groups were grown in serum-free basal media, in parallel to experimental groups. After 24 h, media were removed, the cells were washed once with PBS and total protein was isolated using M-PER reagent (mammalian protein extraction reagent; Thermo Scientific).

Assays were carried out according to the manufacturer’s instructions and performed in triplicate. All experiments were independently repeated three times.

### 2.8. Enzyme-Linked Immunosorbent Assay for Total MSH2 Quantification

We performed a direct enzyme-linked immunosorbent assay (ELISA) to determine the total levels of MSH2 protein expression in NCI and FaDu treated with NNK and their untreated controls, with or without the presence of mimic miR-21 and miR-21 inhibitor. Total protein concentrations were determined using the BCA-200 Protein Assay kit (Thermo Scientific), and total MSH2 expression levels were determined by ELISA as follows:

Nunc MaxiSorp™ 96-well plates (Invitrogen by Thermo Fisher Scientific) were coated with 100 μL of total protein extracts from NCI and FaDu, at a concentration of 10 μg/mL, in 1X coating buffer [protein added to coating buffer and mixed for 15 min; 1X coating buffer diluted from 5X stock (BUF030A; BIORAD) in dH_2_O and mixed for 15 min].

The plates were covered and incubated at 4 °C overnight. The next day, the plates were washed 3 times in wash buffer (PBST; 0.05% v/v Tween-20 in PBS), incubated in 150 μL/well of blocking solution (1% w/v BSA in PBS) for 60 min at 37 °C, and then washed 4 times in wash buffer and incubated in 100 μL/well of primary MSH2 (Clone D-6) mouse monoclonal antibody HRP (horseradish peroxidase) or β-actin (C4) mouse monoclonal antibody HRP (Santa Cruz Biotechnology), which was used as a reference control for protein normalization, for 1 h at 37 °C (we used 0.3 μg of each antibody per 10 μg of total protein; antibodies were diluted in 1%BSA/PBS). Finally, the plates were washed 3 times in wash buffer and incubated in 100 μL/well of TMB Core+ substrate solution (3,3′, 5, 5′-tetramethylbenzidine plus hydrogen peroxide) BUF062C; Bio-Rad) for 30 min at room temperature in the dark. We read the absorbance values immediately at 600 nm using a microplate reader (Sunergy1, BIOTEK; Gen5^TM^ software, BioTek Instruments Inc., Winooski, VT, USA). Protein standards for both MSH2 and β-actin were used by 1:10 serial dilutions of a highly concentrated protein sample that was positive for MSH2.

Assays were carried out according to the manufacturer’s instructions and performed in triplicate. All experiments were repeated three times, independently.

### 2.9. Cell Viability Assay

We performed a cell viability assay, using Cell Titer-Glo^®^ Luminescent Cell Viability Assay (Promega Corp., Madison, WI, USA), to monitor the effect of 1 μM and 2 μM of NNK on NCI and FaDu treated cells, compared to their corresponding untreated controls. The cells were seeded at a density of 5000 cells/well in 24-well plates. The next day, the cells were exposed for 24 h to experimental media. At the end of the treatment, we removed the media and replaced them with serum-free basal media. Untreated controls were also grown in serum-free basal media. Cells were cultured at 37 °C in humidified air and 5% CO2 for 3 days. We then used a luminometer to measure the luminescence. We determined the cell viability by comparing the mean values of cells exposed to NNK versus the mean values of untreated cells, for each cancer cell line. The statistically significant difference in cell viability was determined using paired-test and *p*-value < 0.05 (Graph Pad Prism 7.0, GraphPad Software Inc., San Diego, CA, USA). All experiments were repeated three times, independently.

### 2.10. Statistical Analysis

We used GraphPad Prism 7 software and multiple *t*-test analysis (GraphPad Prism 7 software; *t*-test; multiple comparisons by Holm-Sidak) to show the differential expression (*p*-values) for each analyzed gene and protein expression between different experimental and control groups. Pearson correlation was performed to estimate the correlation coefficient between the transcriptional levels of the analyzed MMR genes and the proteins, as well as between the expression levels of MMR genes or proteins and “oncomirs”, in the different NCI and FaDu groups (*p*-values < 0.05).

## 3. Results

### 3.1. Either Low or High Dose of NNK Reduces MSH2 and MLH1 Protein Levels in Both LSCC and HSCC Cells

**Figure 1:**
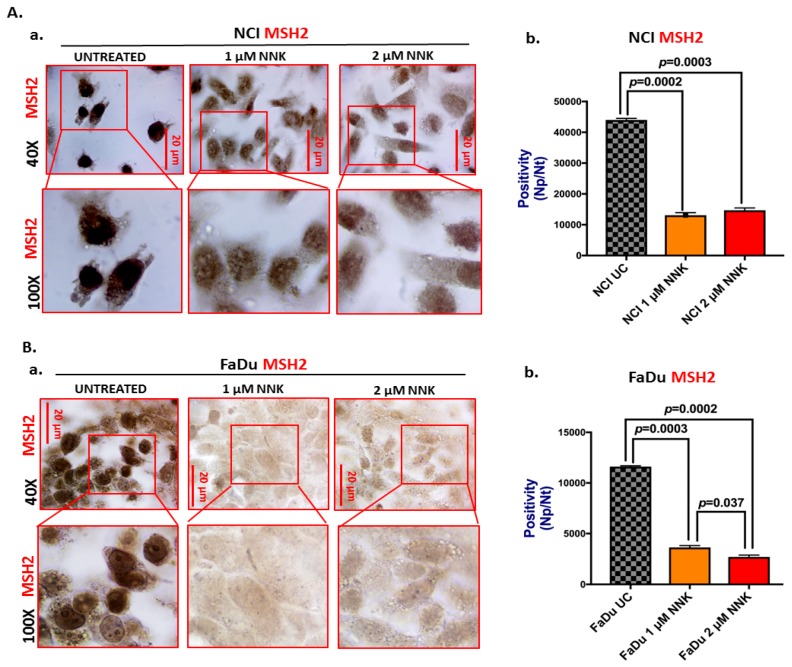
Either low or high dose of NNK reduces MSH2 expression in both lung (NCI) and head and neck (FaDu) cancer cells. Immunoperoxidase cell staining for MSH2 reveals that (**A**) NCI and (**B**) FaDU exposed to either 1 μM or 2 μM of NNK produce reduced MSH2 nuclear levels, as indicated by (**A**-**a** and **B**-**a**) the less intense MSH2 staining (scale bar: 20 μm), and (**A**-**b** and **B**-**b**) the significantly lower nuclear MSH2 levels [Np/Nt = Number of nuclear positive/total number of nuclei, means(SD)], compared to untreated controls. Data were obtained from two independent images (≥10 cells) (*p* values by *t*-test; multiple comparisons by Holm-Sidak; GraphPad Prism 7.0). Images were captured using Aperio CS2 and analyzed by Image Scope software (Leica Microsystems, Buffalo Grove, IL).

**Figure 2:**
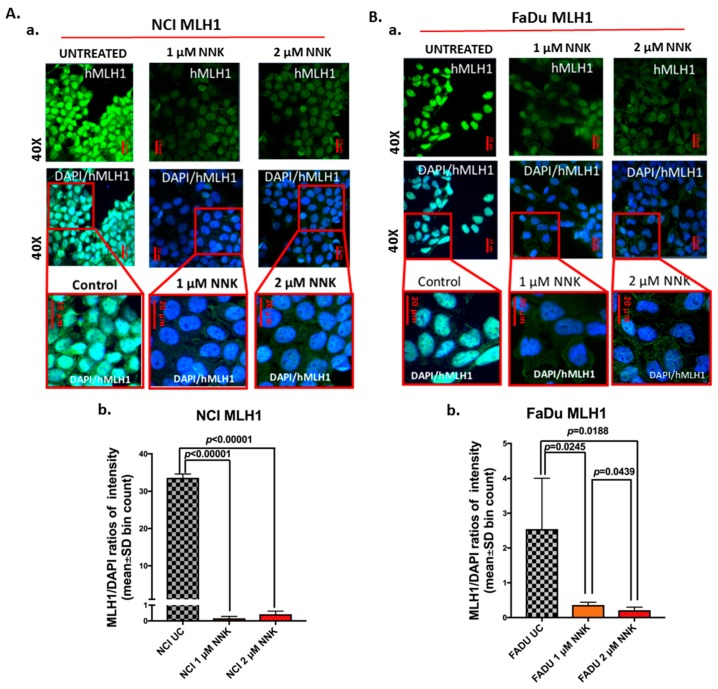
Either low or high dose of NNK reduce MLH1 expression in both lung (NCI) and head and neck (FaDu) cancer cells. Immunofluorescence staining for MLH1 reveals that (**A**) NCI and (**B**) FaDU exposed to either 1 μM or 2 μM of NNK produce reduced MLH1 nuclear levels, as indicated by (**A**-**a** and **B**-**a**) the weak nuclear staining of MLH1 (green: MLH1; blue: DAPI for nuclear staining; scale bar: 20 μm), and (**A**-**b** and **B**-**b**) the significantly lower nuclear MLH1 levels [MLH1/DAPI means(SD) bin count, by Zen imaging software], compared to untreated controls. Data were obtained from four independent images (*p* values by *t*-test; multiple comparisons by Holm-Sidak; GraphPad Prism 7.0).

**Figure 3:**
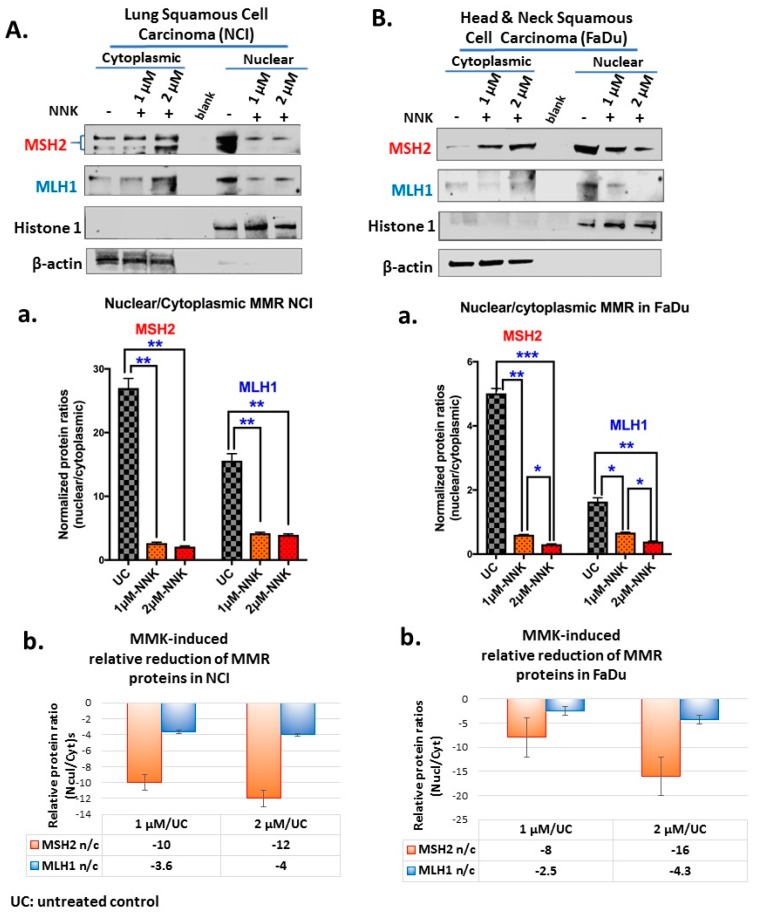
Either low or high dose of NNK reduces the nuclear translocation of MMR (MSH2 and MLH1) proteins in both (**A**) NCI and (**B**) FaDu cells. Graphs depict MSH2 and MLH1 nuclear translocation ratios (nuclear/cytoplasmic protein expression levels) (**A**-**a** and **B**-**a**), in NCI and FaDu cells, respectively, exposed to 1 μM or 2 μM of NNK compared to untreated controls. (**A**-**b** and **B**-**b**) NNK-induced relative reduction of MMR nuclear translocation in NCI and FaDu cells, respectively, is demonstrated by the relative MMR n/c (nuclear/cytoplasmic) protein ratios in NNK-treated vs. untreated controls. (β-actin and Histone 1 were used to normalize cytoplasmic and nuclear protein extracts, respectively, by western blot analysis; UC: untreated controls). [Paired *t*-test, * *p* < 0.05; ** *p* < 0.005; *** *p* < 0.0005; **** *p* < 0.00005; GraphPad Prism 7.0; means (SD) of three independent experiments].

Immunocytochemical and western blot analyses revealed that exposure to either a low or high dose of NNK causes a significant decrease in the expression and nuclear translocation of mismatch DNA repair proteins, MSH2 and MLH1, in both NCI and FaDu treated cells, compared to untreated controls (Figure 1, Figure 2, and Figure 3).

Specifically, as depicted in Figure 2 by immunocytochemical analysis, both untreated NCI and FaDu cells showed strong nuclear MSH2 localization. In contrast, both NCI and FaDu exposed to either a low (1 μM) or high (2 μM) dose of NNK exhibited weak nuclear and/or cytoplasmic staining for MSH2 compared to untreated controls (Figure 1A-a,B-a). Scoring of MSH2 positivity revealed significantly lower MSH2 levels in NCI and FaDu exposed to either 1 μM or 2 μM of NNK, compared to untreated controls (Figure 1A-b,B-b) [*p* < 0.05, *t*-test; means (SD); multiple comparisons by Holm-Sidak].

Also, as depicted in Figure 2, by immunofluorescence assay, both untreated NCI and FaDu cells showed intense nuclear staining of MLH1. In contrast, both NCI and FaDu exposed to either a low (1 μM) or high (2 μM) dose of NNK exhibited weak staining for nuclear MLH1 (Figure 2A-a,B-a). Scoring of nuclear MLH1 revealed statistically significantly lower MLH1 levels in NCI and FaDu exposed to 1 μM or 2 μM of NNK, compared to untreated controls (Figure 2A-b,B-b) [*p* < 0.05, *t*-test; means (SD); multiple comparisons by Holm-Sidak]. Scoring of nuclear MLH1 also revealed statistically significantly lower MLH1 levels in FaDu exposed to 2 μM compared to those exposed to 1 μM of NNK.

Western blot analysis confirmed the above immunohistochemical analysis data. Specifically, as shown in Figure 3, both NCI and FaDu exposed to a low (1 μM) or high (2 μM) dose of NNK showed significantly lower MSH2 and MLH1 nuclear translocation ratios (nuclear vs. cytoplasmic levels) (Figure 3A-a,B-a) compared to untreated controls. In general, the high NNK dose induced lower nuclear translocation ratios of MMR proteins compared to the low dose (Figure 3A-b,B-b). NCI exposed to either a low or high dose of NNK produced a similar reduction of MSH2 and MLH1. In general, total MMR (MSH2 and MLH1) protein levels were significantly reduced in NNK-treated NCI and FaDu, relative to their untreated controls (Supplementary Figure S1).

### 3.2. Either Low or High Dose of NNK Reduces hMSH2 and hMLH1 mRNAs in Both LSCC and HSCC Cells

**Figure 4:**
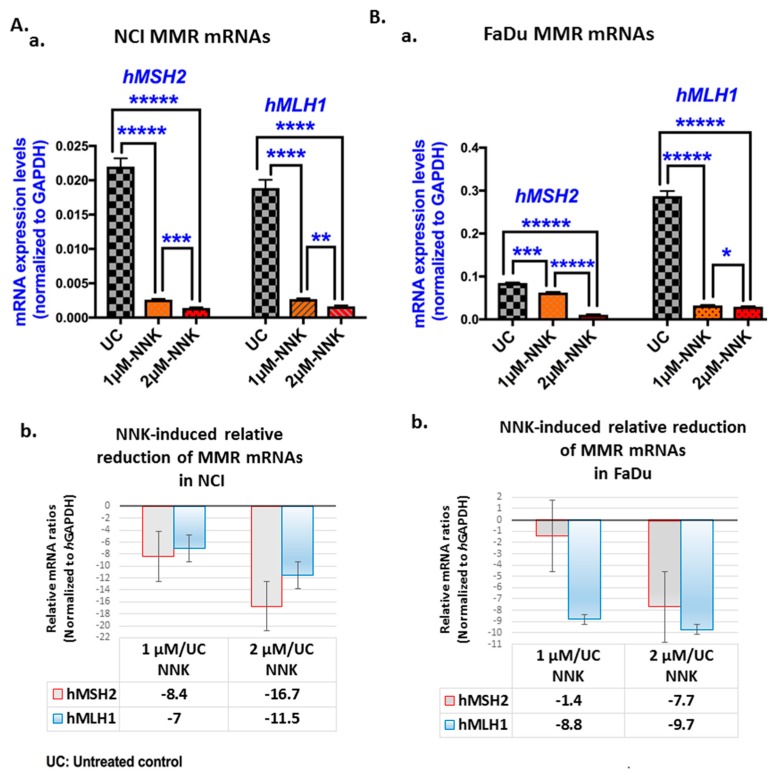
Either low or high dose of NNK reduces *hMSH2* and *hMLH1* mRNAs in both (**A**) NCI and (**B**) FaDu. (**A**-**a** and **B**-**a**) Graphs depict the transcriptional levels of the MMR genes, *hMSH2* and *hMLH1* (relative to *h*GAPDH reference gene) induced in untreated and NNK-treated NCI and FaDu. Data were obtained from real-time qPCR analysis. (Graphs, created by GraphPad Prism 7 software; *** *p* < 0.0005; **** *p* < 0.0005, ***** *p* < 0.00005; by *t*-test; multiple comparisons by Holm-Sidak). **(A**-**b and B**-**b)** The graphs illustrate the NNK-induced mRNA reduction, as demonstrated by the relative mRNA ratios of MMR genes (*hMSH2* and *hMLH1*) in NNK-treated (at 1 μM and 2 μM) NCI and FaDu, respectively, versus untreated controls. (Data were obtained from three independent experiments).

Gene expression analysis by quantitative PCR revealed that exposure to either a low or high dose of NNK induced a significant decrease in both *hMSH2* and *hMLH1* mRNAs in treated NCI and FaDu cell lines compared to untreated controls, as illustrated in Figure 4.

Specifically, we found that both NCI and FaDu exposed to 2 μM of NNK produced significantly lower transcriptional levels of *hMSH2* and *hMLH1* compared to those exposed to 1 μM (Figure 4A-a,B-a; Supplementary Table S3).

In general, the high dose of NNK caused a more pronounced decrease in MMR mRNAs compared to the low dose, especially for *hMSH2* (Figure 4A-b,B-b). As shown in Table 1, *hMSH2* showed a 2-fold and 5.7-fold decrease in its mRNAs, in NCI and FaDu cells, respectively, exposed to 2 μM of NNK, as compared to those exposed to 1 μM.

Pearson analysis revealed a significant positive correlation between NNK-induced *hMSH2* and *hMLH1* reduced mRNA levels in NCI (*r* = 0.9999, *p* < 0.009). A positive correlation was also found between NNK-induced *hMSH2* and *hMLH1* mRNAs in FaDu; however, this was not found to be statistically significant (*r* = 0.74).

Pearson analysis also revealed a significant positive correlation between NNK-induced depleted *hMSH2* mRNAs and MSH2 nuclear protein levels in NCI (*r* = 0.9997, *p* = 0.0155). Positive correlations were also found between NNK-induced decreased *hMLH1* mRNAs and MLH1 nuclear protein levels in both NCI (*r* = 0.9798) and FadU (*r* = 0.9458); however, these were not found to be statistically significant.

The above observations support a linear correlation between the two main components (MSH2 and MLH1) of the MMR mechanism in both NCI and FaDu and between the levels of mRNAs and proteins of each MMR component in upper-aerodigestive tract cancer cells under NNK exposure.

### 3.3. NNK Induces Deregulation of “Oncomirs” miR-21 and miR-155, and “Tumor Suppressor” miR-422a, in Exposed LSCC and HSCC Cells

**Figure 5:**
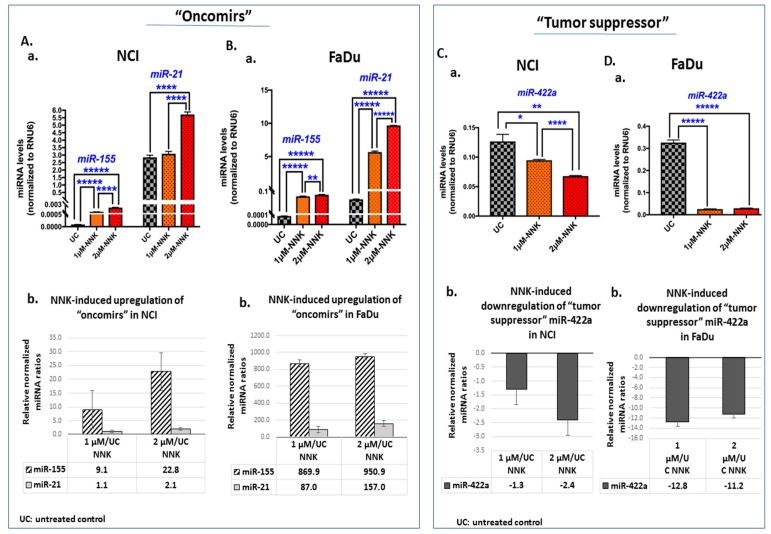
Either low or high dose of NNK induces deregulation of “oncomirs”, miR-21 and miR-155, and “tumor suppressor” miR-422a, in (**A**) NCI and (**B**) FaDu. (**A**-**a** and **B**-**a**) Graphs depict the expression levels of “oncomirs”, miR-155 and miR-21, in untreated and NNK-treated NCI and FaDu, respectively. (**A**-**b** and **B**-**b**) The graphs illustrate the NNK-induced upregulation of “oncomirs”, miR-21 and miR-155, in NCI and FaDu cells, respectively, as demonstrated by the relative expression ratios in NNK-treated (1 μM or 2 μM) versus untreated controls. (**C**-**a** and **D**-**a**) The graphs illustrate the NNK-induced downregulation of “tumor suppressor” miR-422a, in NCI and FaDu, respectively, as demonstrated by the relative expression ratios in NNK-treated (1 μM or 2 μM) versus untreated controls. [miRNA levels were normalized to RNU6 reference control. Data were obtained from three independent experiments; *p* values by *t*-test; * *p* < 0.05, ** *p* < 0.005, **** *p* < 0.00005, ***** *p* < 0.000005; GraphPad Prism 7.0, means (SD)].

MicroRNA analysis revealed that either a low or high dose of NNK can upregulate the “oncomirs”, miR-155 and miR-21 (Figure 5A,B), and downregulate the “tumor suppressor” miR-422a (Figure 5C,D) in NCI and FaDu, respectively.

We found that both NCI and FaDu exposed to 2 μM of NNK produced significantly higher miR-21 and miR-155 levels compared to cells exposed to 1 μM (Figure 5A-a,B-a). We also found that NCI exposed to 2 μM of NNK produced significantly lower levels of miR-422a compared to those exposed to 1 μM (Figure 5C-a,D-a) (Supplementary Table S4).

In general, the high dose of NNK caused a more profound upregulation of the analyzed “oncomirs” compared to the low dose, especially for miR-21, in both NCI and FaDu (Figure 5A-b,B-b). As shown in Table 1, miR-21 showed a 1.8-fold and 2.0-fold increase in NCI and FaDu, respectively, and miR-155 showed a 2.5-fold increase in NCI exposed to 2 μM of NNK compared to those exposed to 1 μM. On the other hand, the high dose of NNK induced a more profound downregulation of miR-422a than the low dose, in NCI (Figure 5C-b), by a 1.8-fold decrease (2 μM vs. 1 μM of NNK), as shown in Table 1. However, a similar downregulation of miR-422a was found in FaDu exposed to either a low (1 μM) or high (2 μM) dose of NNK (Figure 5D-b).

A Pearson analysis revealed (i) a linear correlation between the levels of miR-21 and miR-155 induced by NNK in both NCI (*r* = 0.9393) and FaDu (*r* = 0.9540), and (ii) an inverse correlation between the levels of miR-155 and miR-422a induced by NNK in both NCI (*r* = −0.9806) and FaDu (*r* = −0.9962), although this was not found to be statistically significant.

### 3.4. Inhibition of miR-21 prevents NNK-induced MSH2 reduction

**Figure 6:**
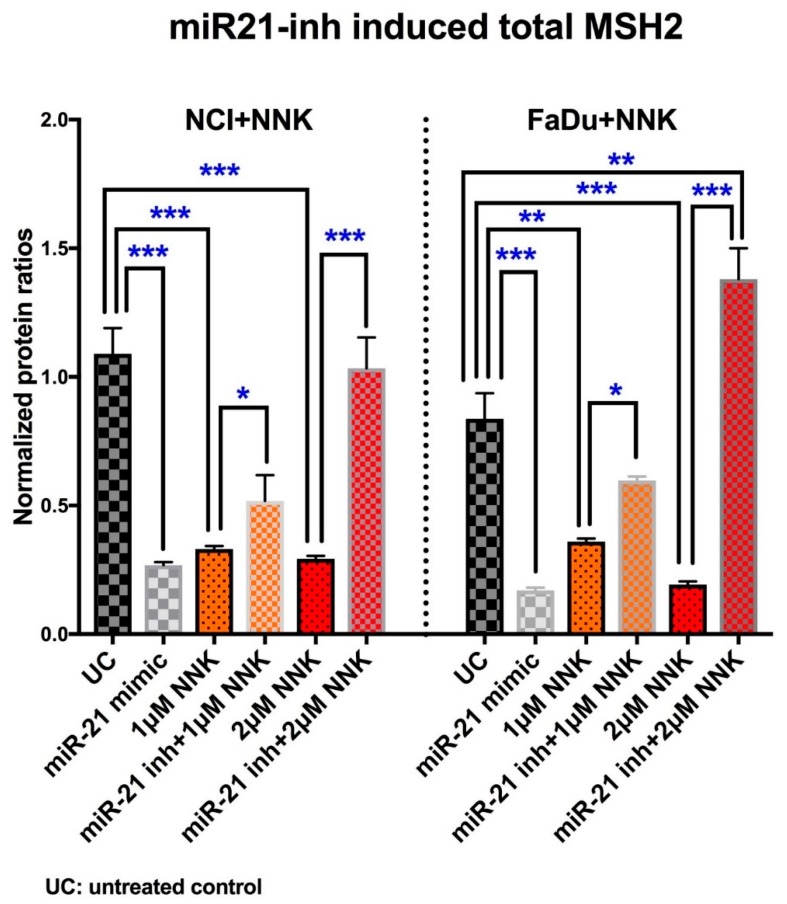
Inhibition of miR-21 prevents NNK-induced MSH2 reduction. Graphs created by GraphPad Prism 7.0 depict total MSH2 protein level in NCI and FaDu exposed to 1 μM or 2 μM of NNK, with and without miR-21 inhibitor. Controls: cells treated with mimic miR-21 and untreated controls. (Data obtained from three independent experiments [* *p* < 0.05; ** *p* < 0.005; *** *p* < 0.0005, by *t*-test; multiple comparisons by Holm-Sidak; GraphPad Prism 7.0; means (SD)].

We used a mimic miR-21 and an inhibitor of miR-21 to mechanically analyze their effect on NNK-induced suppression of total MSH2 protein levels. Elisa revealed that inhibition of miR-21 prevents the reduced MSH2 phenotype induced by NNK in NCI and FaDu treated cells (Figure 6).

We observed that either a low (1 μM) or high (2 μM) dose of NNK caused a significant decrease in total MSH2 protein levels, in both NCI and FaDu treated cells compared to untreated controls. However, inhibition of miR-21 prevented the NNK-induced MSH2 depletion in treated cells (Figure 6, Supplementary Table S5). Specifically, at either low or high doses of NNK, both NCI and FaDu with inhibited miR-21, produced significantly higher levels of MSH2 protein compared to those without miR-21 inhibition. The observation that exposure to a high dose of NNK resulted in a significantly higher MMR expression in cells with inhibited miR-21 compared to untreated controls is probably due to the increased need for DNA repair under the harmful effect of NNK.

The suppressive effect of miR-21 on MSH2 protein levels was demonstrated by the observation that the mimic miR-21 induced significantly lower total MSH2 levels in both NCI and FaDu treated cells than untreated controls.

### 3.5. NNK Increases LSCC and HSCC Cell Viability that Can Be Prevented by miR-21 Inhibition

**Figure 7:**
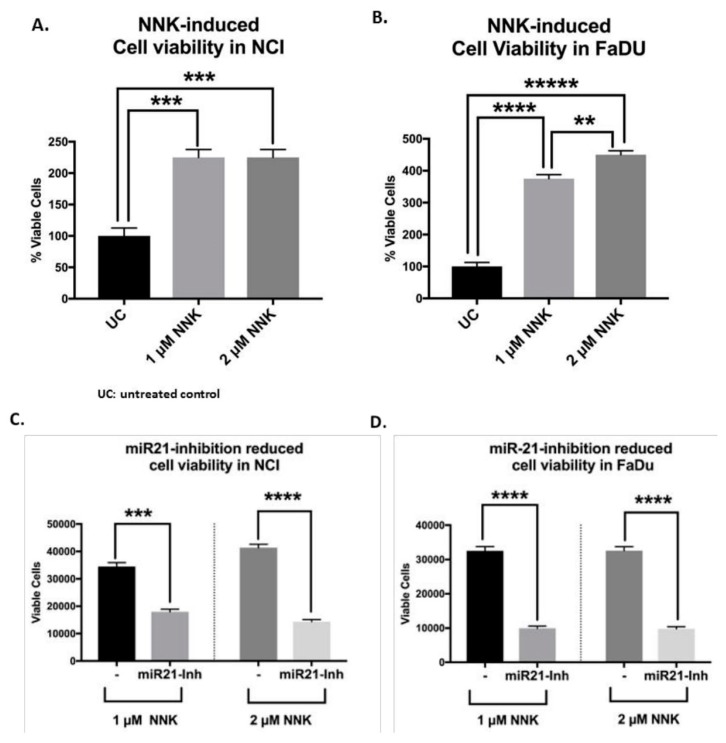
NNK promotes cell viability in lung (NCI) and head and neck (FaDu) squamous cancer cells, which is prevented by miR-21 inhibition. Graphs depict the viability rates in (**A**) NCI and (**B**) FaDu exposed to 1 μM and 2 μM of NNK (% of viable cells in NNK groups vs untreated controls) and the changes in the viability of (**C**) NCI and (**D**) FaDu cells under the inhibition of miR-21 (miR21-inh). Graphs, created by GraphPad Prism 7 software; ** *p* < 0.005; *** *p* < 0.0005; **** *p* < 0.00005; ***** *p* < 0.000005, by *t*-test; multiple comparisons by Holm-Sidak). (Data are derived from three independent experiments.).

NNK increased cell viability in both NCI and FaDu treated cells compared to untreated controls (Figure 7A).

In particular, NCI exposed to 1 μM or 2 μM of NNK exhibited a similar increase in cell viability compared to untreated controls (Figure 7A). Although FaDu exposed to both 1 μM and 2 μM of NNK increased cell viability compared to untreated controls, it appeared that 2 μM of NNK induced significantly higher survival rates compared to 1 μM, in FaDu (Figure 7B).

The contribution of miR-21 in NNK-induced cell viability of NCI and FaDu cells was explored by the application of miR-21 inhibitor. As shown in Figure 7C,D, the application of mi-R21 inhibitor produced a significant reduction in cell viability of NNK-treated NCI and FaDu, respectively) (*p* < 0.0005).

### 3.6. LSCC Versus HSCC under NNK Exposure

NNK induced a more profound effect on miR-21, miR-155 and miR-422a deregulation in HSCC (FaDu) compared to LSCC (NCI) cells, either at a low or high dose. On the other hand, NNK induced a more profound effect on MMR genes downregulation in LSCC compared to HSCC cells, especially at a high dose (Figure 4A-b,B-c).

However, NNK produced a reduced MMR phenotype and miRNA deregulations in both LSCC and HSCC cells. In addition, miR-21 inhibition prevented NNK-induced MSH2 reduction in both LSCC and HSCC cells.

## 4. Discussion

**Figure 8:**
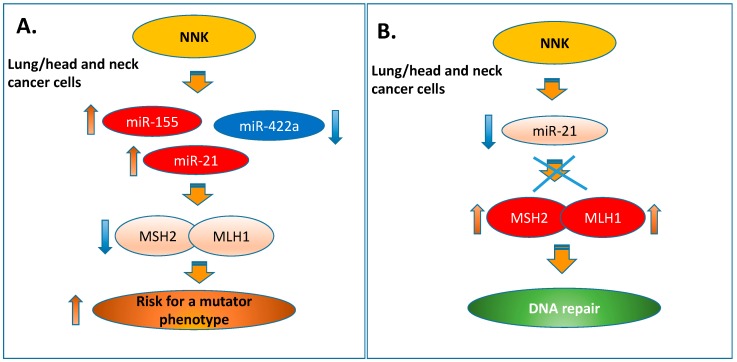
NNK-induced deregulation of miRNA and Mismatch DNA repair expression profiles in lung and head and neck squamous cancer cells. Schematic presentation of (**A**) the proposed mechanism by which NNK induced deregulation of miRNA and MMR genes increasing the risk for a mutator phenotype in lung and head and neck squamous cancer cells (**B**) the proposed preventive effect of miR-21 inhibition in this process.

Tobacco smoke is an established risk factor of lung and head and neck cancers [61,62], and is considered to contribute to their recurrence and progression [63,64]. There is evidence that patients with head and neck squamous cell carcinoma often develop primary squamous cell lung carcinoma [65]. This suggests that common mechanisms may apply in both types of cancer [66]. Here, we demonstrate that tobacco smoke carcinogen, NNK, can affect the expression of cancer-related miR-21, miR-155 and miR-422a, which have a regulatory role in the MMR mechanism, causing downregulation of *hMSH2* and *hMLH1*, and increase cell survival in both lung and head and neck squamous cancer cells (Figure 8A).

In general, the deregulation of microRNA expression has been associated with gene alterations that are commonly linked to smoke-related cancers [67,68,69]. The “oncomirs” miR-21 and miR-155 have previously been associated with cancers of the upper aerodigestive tract [48,49,51,52,53,54]. In particular, miR-21 has been considered as a marker of poor prognosis in lung and head and neck cancer [70,71]. To our knowledge, we are the first to present an association between the NNK effect and miR-21, miR-155 and miR-422a deregulations in lung and head and neck squamous cancer cells, supporting them as possible biomarkers in NNK-induced gene alterations.

In principle, miRNAs are involved in the regulation of numerous genes related to various physiological processes, including MMR genes [38,39,40,41,42,43,44,45,46,72]. As we previously discussed, miR-155 and miR-422a are known to be involved in MMR mechanism regulation [43,46]. Although miRNA-21 is a crucial factor for both types of malignancies, there is limited literature regarding the involvement of miR-21 in the MMR mechanism [39,40,41,42]. Here, our novel findings have shown that the NNK-induced upregulation of “oncomir” miR-21 directly affects MSH2 protein levels in both lung and head and neck squamous cancer cells, and that its inhibition can restore the MSH2 expression phenotype (Figure 8B). These findings document the regulatory role of miR-21 in the MMR mechanism by directly affecting MSH2, which is a key component of the MutSa complex that recognizes base–base mismatches and short insertion and deletion loops [24]. We also showed that inhibition of miR-21 significantly decreases NNK-induced cell survival. Although multiple mechanisms could be involved in this process, our data suggest that the miR-21 could possible play an important role in NNK-induced antiapoptotic effect. This is consistent with previous report suggesting that miR-21 is directly involved in cell cycle regulation by inhibiting MSH2 [42].

NNK is one of the tobacco products that has been evaluated by the International Agency for Research on Cancer (IARC) [73]. Evidence of NNK carcinogenicity was provided by preclinical studies [62]. NNK can be metabolically activated to intermediates that react with DNA forming covalently-bound products known as adducts. The NNK-mediated formation of DNA adducts is crucial to the carcinogenic process [74]. The defect in the DNA repair mechanism leads to mutation due to unrepaired NNK-induced DNA adducts. Although damaged or mutated DNA can be removed by apoptosis, cell survival due to either upregulation of the antiapoptotic mechanism or mutations that occur in cancer-related regions may result in uncontrolled cellular growth (antiapoptotic process) and tumor development. Previous experimental studies provided evidence that MMR deficiency is associated with tumor progression [75]. Our data showed that NNK, especially at a high dose, in parallel to inducing MMR deregulation, also promotes the cellular viability of NCI and FaDu. Clearly, promoting the antiapoptosis in a cell with a defective DNA repair mechanism increases the risk for a mutator phenotype (Figure 8A), and potentially increases the risk for malignant progression.

The role of NNK-induced tumorigenicity appeared to be complex. Earlier studies have shown that NNK-induced decreased binding of the nucleotide excision repair (NER) proteins XPA and XPB to DNA could be responsible for the decrease in repair activities in lung [76,77]. This observation supports the hypothesis that NNK can not only alter the level of MMR proteins, but may, in parallel, affect modifications of NER proteins, resulting in defective DNA repair. It is believed that NNK can affect the level of proteins related to DNA repair mechanisms by inducing the formation of pyridyloxobutyl and methyl adducts [77]. Specifically, NNK-induced DNA methylation, like promoter hyper-methylation of MLH1 and MSH2 [78,79], can significantly affect the ability of cells to repair genetic damage [80]. Therefore, the observed NNK-induced alterations of the MMR gene expression presented here cannot rule out their epigenetic regulation through the NNK-induced DNA methylation. However, NNK-induced DNA methylation can, at the same time, modulate miRNA levels by regulating MMR gene expression. Hypo- or hyper- methylation of miRNA was considered to represent a new level of complexity in gene regulation in human cancers [81], suggesting miR-21 or miR-155 promoter hypo-methylation [81,82,83,84] and miR-422a hyper-methylation, as previously reported for miR-373 [81,85], as potential epigenetic modifications caused by tobacco carcinogenic effects on MMR. On the other hand, alkylating agents, such as NNK can also directly or after biological activation react and form covalent bonds with nucleophilic centers found in DNA and RNA and proteins [86], supporting possible direct interference of NNK with levels of miRNAs, thereby causing their deregulation [48,49,51,52,53,54]. Subsequently, NNK-induced miRNA deregulations can affect MMR gene expression, either thought post-transcriptional modifications or through DNA methylation by targeting DNA methyltransferases or methylation-related proteins [81].

Our novel findings showed that either a low or high NNK dose can cause a significant upregulation of “oncomirs” miR-21 and miR-155 and downregulation of “tumor suppressor” miR-422a, as well as a decrease in *hMSH2* and *hMLH1* at both transcriptional and protein expression levels in exposed lung and head and neck squamous cancer cells. Although further exploration of a possible dose-dependent effect of NNK on the MMR mechanism is required, our study showed that a higher NNK dose induces a more extended effect on miRNA, particularly on miR-155 and miR-422a, and MMR expression compared to lower doses. On the other hand, the fact that even a low dose of NNK was capable of causing significant alterations in MMR and miRNA expression indicates that even a small exposure to the tobacco smoke carcinogen could have potentially harmful consequences. Taking into consideration the theory that long-term exposure to chemicals, even at low doses, could have an augmented effect [87,88], long-term studies could reveal the chronic effect of low NNK exposure to MMR genes in lung and head and neck squamous cancer cells.

It is thought that high levels of MMR can positively contribute to the efficacy of chemotherapy [89,90], while a significant number of preclinical and clinical data have shown that inactivation of *hMLH1* and *hMSH2* promotes resistance to cisplatin and carboplatin-based chemotherapy [34,35]. According to previous findings from our group, a defective MMR phenotype is not beneficial for cisplatin chemotherapy, resulting in low survival rates in patients with lung squamous cell carcinoma [34]. Recently, there has been increasing interest in assessing the predictive value of a defective MMR mechanism in various types of cancer, including lung and head and neck cancer [91,92]. In addition, recent studies strongly support that MMR status can alter the efficacy of target immunotherapy, and the identification of MMR status prior to initiation of treatment may be a useful approach [36,83]. The fact that NNK, in addition to its mutagenic effect, which is manifested by inducing DNA defects, can simultaneously suppress the DNA repair mechanism and promote cellular antiapoptosis, supports its carcinogenic potency. Our observation that NNK caused a decrease in the expression of MMR genes in lung and head and neck squamous cancer cells may support the theory that exposure of these cells to tobacco smoke could have a potential modulatory effect in the treatment and natural history of the disease.

## 5. Conclusions

In summary, the current study shows that NNK, either at a high or low dose, can cause deregulation in miR-21, miR-155 and miR-422a, and downregulation of MMR genes. In addition to promoting the deregulation of the MMR mechanism, NNK can simultaneously enhance the viability of cancer cells, potentially promoting cancer progression. Finally, inhibition of miR-21 can restore NNK-induced MSH2 reduction and decreases cell survival.

All the above provide further information on the effects of NNK on cancer development and progression, and give insights into the impact that smoke-carcinogens could have on the MMR status. Further studies should reveal the utility of MMR genes and miRNAs as diagnostic biomarkers and as a tool for novel diagnostic and therapeutic approaches in lung and head and neck squamous cell carcinomas.

## Figures and Tables

**Table 1:** MMR mRNA and miRNA expression changes produced in Lung (NCI) and Head and Neck (FaDu) cancer cells exposed to high (2 μΜ) versus to low (1 μΜ) dose of NNK.

Upper Aero-Digestive Tract Cancer Cell Lines	*2 μM vs. 1 μM of NNK
Lung cancer cells (NCI)	
MMR genes	
*hMSH2*	2.0 ↓
*hMLH1*	1.6 ↓
miRNAs	
*miR-155*	2.5 ↑
*miR-21*	2.0 ↑
*miR-422a*	1.8 ↓
Head and Neck cancer cells (FaDu)	
MMR genes	
*hMSH2*	5.7 ↓
*hMLH1*	1.1 ↓
miRNAs	
*miR-155*	1.1 ↑
*miR-21*	1.8 ↑
*miR-422a*	1.0

* Fold-changes in normalized mRNA and miRNA levels (↓: decrease; ↑: increase)

## Supplementary Materials

The data supports the findings of this study are available in the supplementary material of this article at https://www.mdpi.com/2073-4409/9/4/1031/s1 (Table S1: Human genes analyzed by real-time qPCR, in human lung (NCI) and head and neck (FaDu) cancer cells; Table S2: Human miRNAs and small RNA analyzed by real-time qPCR, in human lung (NCI) and head and neck (FaDu) cancer cells; Table S3: Transcriptional levels of MMR genes in human lung (NCI) and head and neck (FaDu) cancer cells (by qPCR); Table S4: Expression levels of miRNA specific markers analyzed in human lung (NCI) and head and neck (FaDu) cancer cells (by qPCR); Table S5: Total MSH2 protein level in NCI and FaDu exposed to 1 μM or 2 μM of NNK, with and without miR-21 inhibitor and Figure S1: Total MMR protein levels in NNK treated NCI and FaDu.
