# Supplementary material for: The Effect of NNK, A Tobacco Smoke Carcinogen, on the miRNA and Mismatch DNA Repair Expression Profiles in Lung and Head and Neck Squamous Cancer Cells
Source: Cells. 2020 Apr 21;9(4):1031. doi: 10.3390/cells9041031 (PMC7226174; doi:10.3390/cells9041031)
Supplement: Supplementary file 1 [file cells-09-01031-s001.pdf]

cells
MDPI

Supplementary Material

The Effect of NNK, A Tobacco Smoke Carcinogen, on the miRNA and Mismatch DNA Repair Expression Profiles in Lung and Head & Neck Squamous Cancer Cells

Sotirios G. Doukas [1,2], Dimitra P. Vageli [2,*], George Lazopoulos [3], Demetrios A. Spandidos [4], Clarence T. Sasaki [2] and Aristidis Tsatsakis [1]

[1] Department of Forensic Sciences and Laboratory of Toxicology, Medical School, University of Crete, 71003 Heraklion, Greece; sotirios.doukas.mail@gmail.com (S.G.D.); tsatsaka@uoc.gr (A.T.)
[2] Department of Surgery, The Yale Larynx Laboratory, New Haven, CT 06510, USA; dimitra.vangeli@yale.edu (D.P.V.); clarence.sasaki@yale.edu (C.T.S.)
[3] Department of Cardiothoracic Surgery, Medical School, University of Crete, 71110 Heraklion, Greece; g.lazopoulos@med.uoc.gr
[4] Laboratory of Clinical Virology, Medical School, University of Crete, 71110 Heraklion, Greece; spandidos@spandidos.gr
* Correspondence: dimitra.vangeli@yale.edu; Tel.: (203) 737 1447

Supplementary Table S1: Human genes analyzed by real-time qPCR, in human lung (NCI) and head and neck (FaDu) cancer cells.

Gene	Detected transcripts	Amplicon length (bp)
hGAPDH	NM_001256799, NM_002046	95
Hs_MSH2	NM_000251	86
Hs_MLH1	NM_000249,<br>NM_001167618-9,<br>NM_001258271-4,<br>XM_005265161,3,4,6	127

Supplementary Table S2: Human miRNAs and small RNA analyzed by real-time qPCR, in human lung (NCI) and head and neck (FaDu) cancer cells.

miRNA (human)	Target mature miRNA (Sanger Accession)
miR-21	hsa-miR-21-5p, MIMAT0000076
miR-155	hsa-miR-155-5p, MIMAT0000646
miR-422a	hsa_miR-422a-5p, MIMAT0001339
Syn-hsa-miR-21-5p	HSA-MIR-21-5P, MIMAT0000076
Anti-hsa-miR-21-5p	HSA-MIR-21-5P, MIMAT0000076
**Small RNA**	**Control**
RNU6-2 (RNU6-6P RNA, U6 small nuclear 6, pseudogene)	Hs_RNU6-2_11

Supplementary Table S3. Transcriptional levels of MMR genes in human lung (NCI) and head and neck (FaDu) cancer cells (by qPCR).

A. NCI.

**Target gene/ *hGapdh** (*ΔΔ*^{CT})**	**NCI untreated**	**NCI 1μM**	**NCI 2μM**
hMSH2	2.20E-02	2.61E-03	1.32E-03
hMLH1	1.89E-02	2.69E-03	1.64E-03

* normalization of mRNA levels using *hGapdh*

B. FaDu.

**Target gene/ *hGapdh**(*ΔΔ*^{CT})**	**FaDu untreated**	**FaDu 1μM**	**FaDu2μM**
hMSH2	8.49E-02	6.25E-02	1.10E-02
hMLH1	2.87E-01	3.26E-02	2.95E-02

* normalization of mRNA levels using hGapdh

Supplementary Table S4. Expression levels of miRNA specific markers analyzed in human lung (NCI) and head and neck (FaDu) cancer cells (by qPCR).

A. NCI.

**Target miRNA/ *RNU6** (*$\Delta\Delta^{CT}$*)**	**NCI untreated**	**NCI 1μM**	**NCI 2μM**
miR-21	2.86E + 00	3.07E + 00	5.98E + 00
miR-155	9.00E-05	8.20E-04	2.05E-03
miR-422a	1.27E-01	9.46E-02	5.30E-02

* normalization of miRNA levels using RNU6

B. FaDu.

**Target miRNA/ *RNU6**(*$\Delta\Delta^{CT}$*)**	**FaDu untreated**	**FaDu 1μM**	**FaDu2μM**
miR-21	6.36E-02	5.57E + 00	9.97E + 00
miR-155	8.90E-05	7.74E-02	8.46E-02
miR-422a	3.26E-01	2.56E-02	2.91E-02

* normalization of miRNA levels using RNU6

Supplementary Table S5. Total MSH2 protein level in NCI and FaDu exposed to 1 μM or 2 μM of NNK, with and without miR-21 inhibitor.

	**Untreated control**	**mimic miR21**	**1 μM NNK**	**miR-inh + 1 μM NNK**	**2 μM NNK**	**miR-inh + 2 μM NNK**
**NCI** MSH2/β-actin	1.09E + 00	2.68E-01	3.31E-01	5.18E-01	2.93E-01	1.03E + 00
**FaDu** MSH2/β-actin	8.37E-1	1.70E-01	4.50E-01	5.98E-01	1.92E-01	1.38E + 00

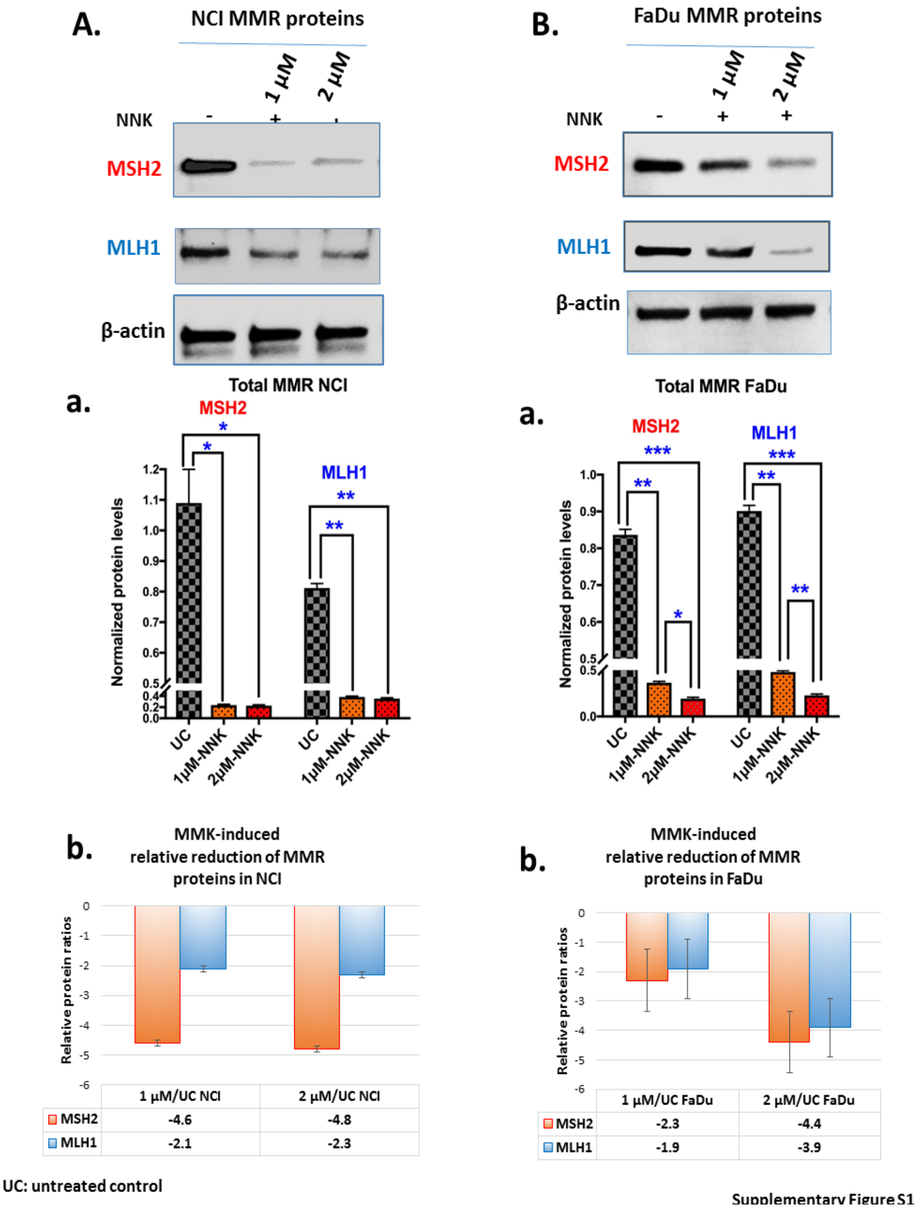

Supplementary Figure S1. Either low or high dose of NNK reduces the total MMR (MSH2 and MLH1) protein levels in both (**A**) NCI and (**B**) FaDu. Graphs depict MSH2 and MLH1 total levels **(A-a** & **B-a)** and NNK-induced relative reduction of total MMR proteins (expression ratios in NNK-treated vs untreated controls) **(A-b** & **B-b)**, in NCI and FaDu cells, respectively. (β-actin was used to normalize total protein extracts, by western blot analysis; UC: untreated controls). [Paired t-test, * $p < 0.05$; ** $p < 0.005$; *** $p < 0.0005$; cells exposed to 1 μM or 2 μM of NNK compared to untreated controls, by western blot analysis. [Paired t-test, GraphPad Prism 7.0; means(SD) of three independent experiments].
